# Supplementary material for: GPR182 limits antitumor immunity via chemokine scavenging in mouse melanoma models
Source: Nat Commun. 2022 Jan 10;13:97. doi: 10.1038/s41467-021-27658-x (PMC8748779; doi:10.1038/s41467-021-27658-x)
Supplement: Supplementary file 1 — Supplementary Information [file 41467_2021_27658_MOESM1_ESM.pdf]

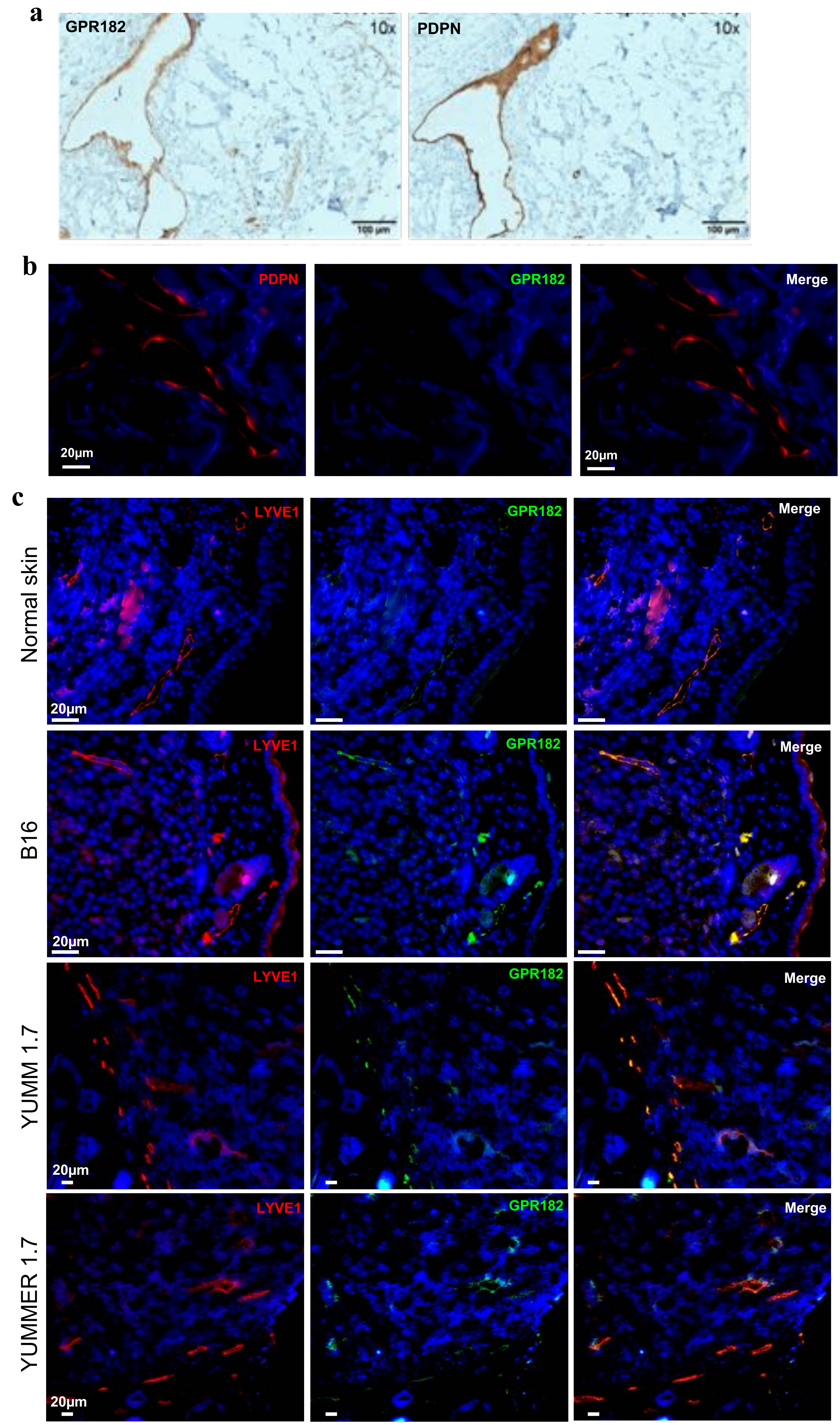

Supplementary Figure 1 GPR182 is primarily expressed in lymphatics within tumors.
(**a**) Immunohistochemistry staining of human breast cancer for GPR182 and PDPN in serial tissue sections. Representative data from two independent experiments.
(**b**) Human normal skin was costained for podoplanin (PDPN) and GPR182. Representative data from two independent experiments.
(**c**) Normal mouse skin, B16 , YUMM1.7 and YUMMER1.7 tumors from WT B6 mice were co-stained for mouse GPR182 and LYVE-1. Representative data from three independent experiments.

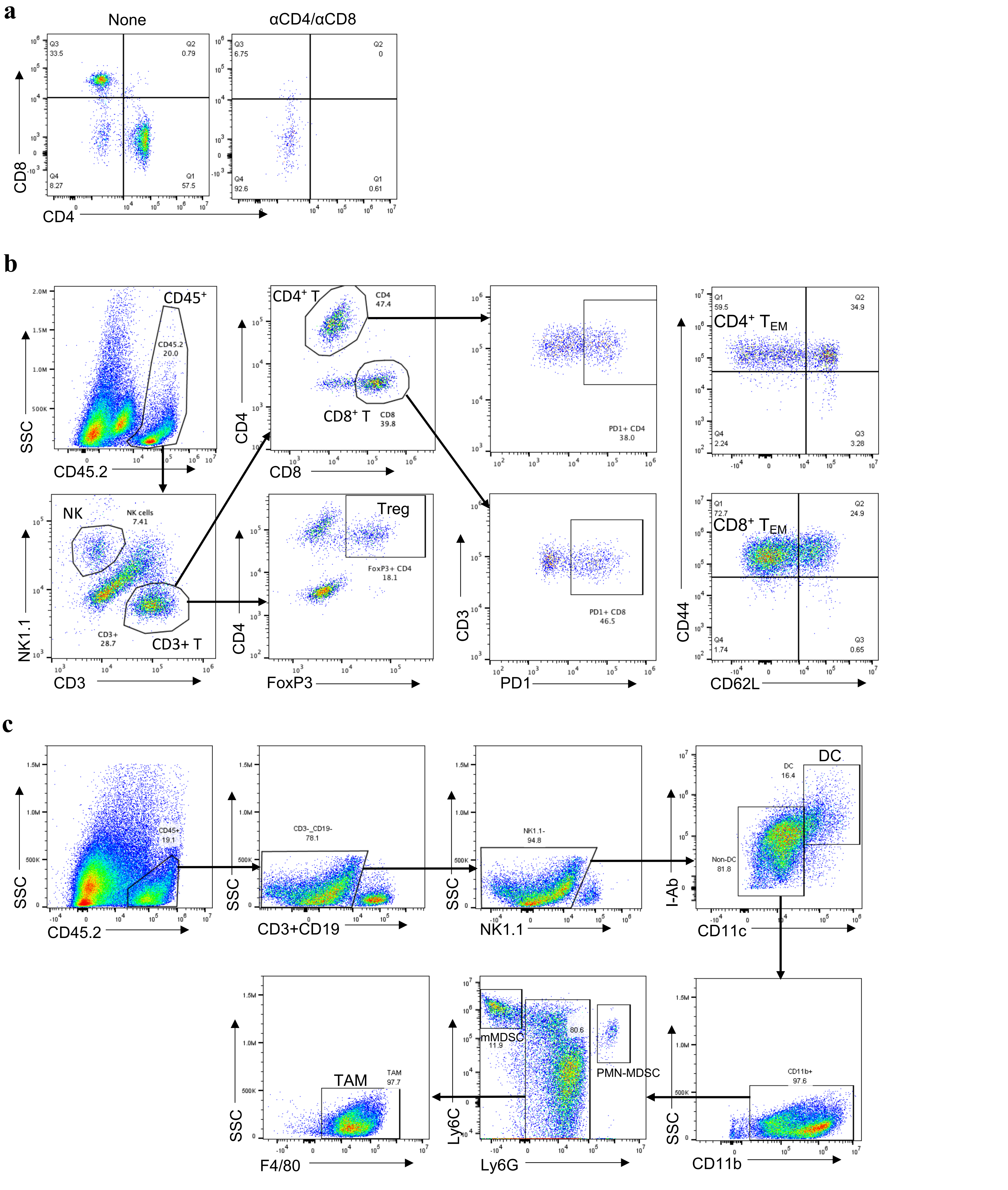

Supplementary Figure 2 Immune cell flow cytometry gating.

(**a**) Confirmation of CD4+ and CD8+ T cell depletion by flow cytometry staining of peripheral blood immune cells after gating on live/CD45+/CD3+/singlets (Fig. 2H).

(**b**) Detailed flow cytometry analysis for tumor- infiltrating lymphocytes in YUMM1.7 tumors after gating on live/singlets: NK (CD45+CD3-NK1.1+), Treg (CD45+CD3+CD4+FoxP3+), T_{EM} ($CD44^{high}$ CD62L-).

(**c**) Gating strategy for myloid-derived immune cells in tumors. Tumor-associated macrophage (TAM, CD45+CD3-NK1.1-CD11c-CD11b+F4/80+), DC (CD45+CD3-NK1.1-CD11c+I-Ab+), monocytic MDSC (mMDSC, CD11c-CD11b+Ly6C+), polymorphonucler MDSC (PMN-MDSC, CD11c-CD11b+Ly6G+).

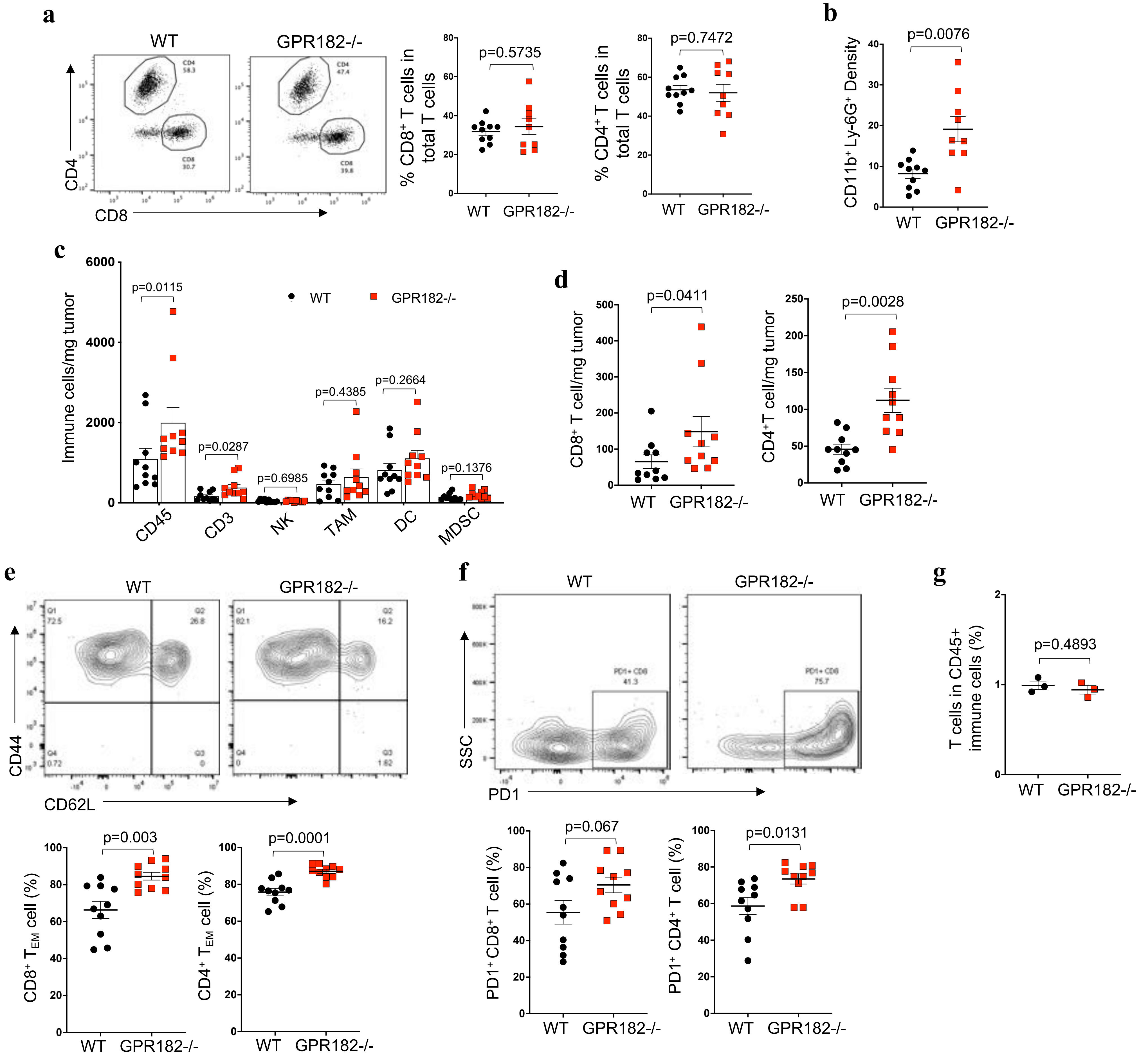

Supplementary Figure 3 Tumors in GPR182-/- mice exhibited increased T cell infiltration and activation

(**a**, **b**) CD3+ T cells in YUMM1.7 tumors (**Fig. 3A**) were further examined for CD4+ and CD8+ T cell subsets (**a**). The densities of PMN-MDSC within YUMM1.7 tumors were quantified by flow cytometry (**b**). n=9-10 per group.

(**c-f**) Tumor infiltrating immune cells were quantified by flow cytometry demonstrating increased CD45+ and CD3+ density in B16 tumors from GPR182-/- tumors compared to WT controls (**c**). The densities of CD8+ and CD4+ T cells within tumors were quantified (**d**) . Tumor infiltrating T cells were stained for CD44 and CD62L to determine the frequencies of effector memory CD8+ T cells (T_{EM}: CD44+CD62L-) (**e**). Tumor infiltrating T cells were stained for PD1 to determine the frequencies of PD1+ T cells within tumors (**f**). n=9-10 per group; two technical replicates.

(**g**) Single cell suspensions were made from normal skin of WT and GPR182-/- mice to determine the percentages of CD3+ T cells in CD45+ immune cells by flow cytometry. n=3 per group.

P-values from two-sided Student's t-test (**a-g**). Error bars represent SEM (**a-g**). All data representative data from two independent experiments.

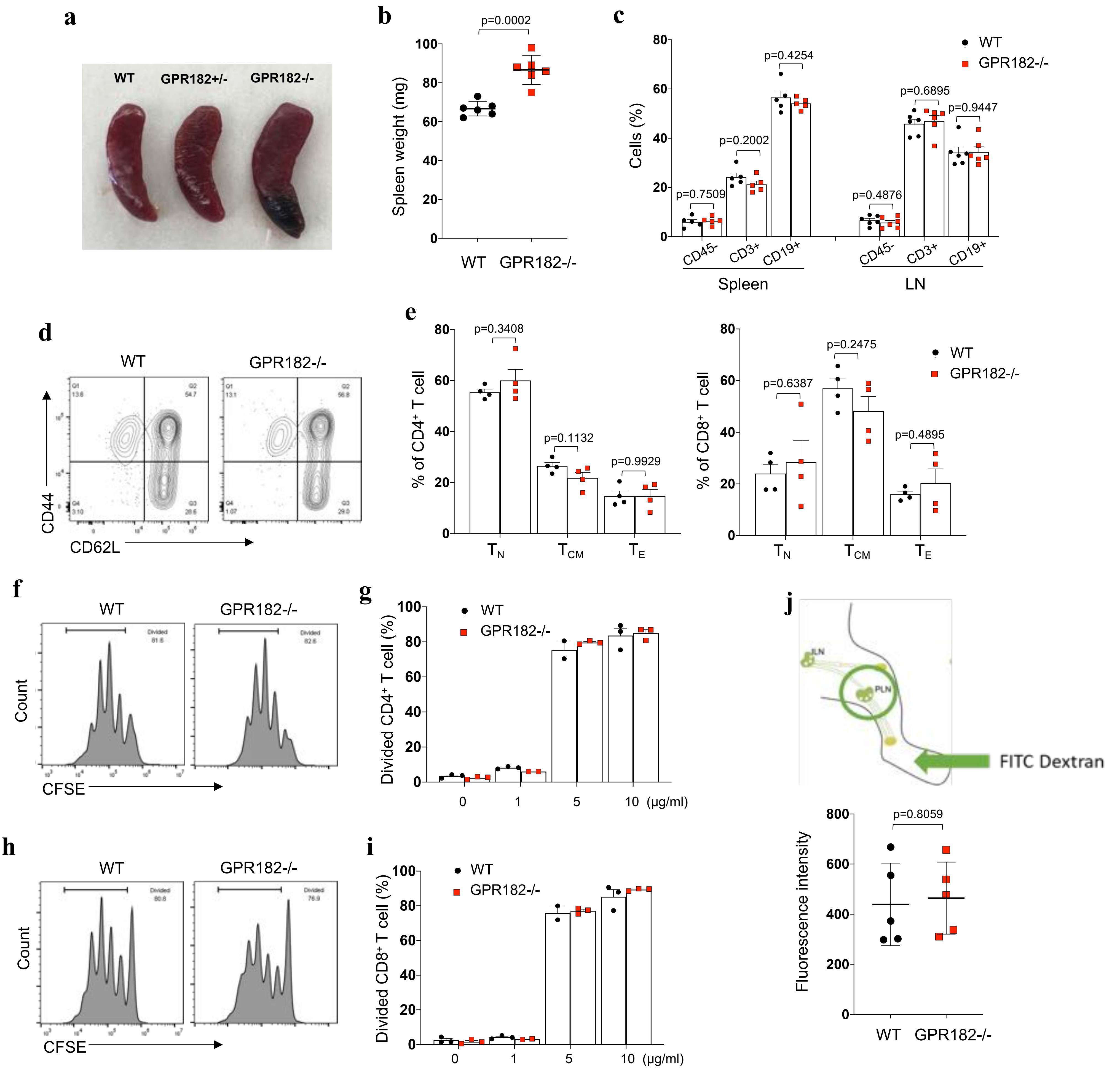

Supplementary Figure 4 Immunophenotyping GPR182-/- mice

(**a**) Representative images of spleens from 10- week- old naïve WT, GPR182+/-, and GPR182-/- mice and (**b**) spleen weights from WT and GPR182-/- mice. n=6 per group.

(**c**) Spleen and lymph node were harvested from WT and GPR182-/- for flow cytometric comparison of immune populations. n=3 per group.

(**d**, **e**) Peripheral blood CD4+ and CD8+ T cells were stained for CD44 and CD62L to characterize naïve (T_N), central memory (T_{CM}), and effector memory (T_{EM}) populations between 10- week- old naïve WT and GPR182-/- mice. Representative flow cytometry plots of CD8+ T cells (D) and quantification (E). n=5 per group.

(**f-i**) Single cell suspensions prepared from lymph nodes of WT and GPR182-/- mice were labelled with CFSE and stimulated *in vitro* with plate bound anti-CD3 for 72 hours. T cell division was quantified by flow cytometry. Representative flow cytometry histograms of CFSE- labelled CD4+ and CD8+ T cell division at 5 µg/ml anti-CD3 (**f** and **h**, respectively) and quantification of divided CD4+ and CD8+ T cell percentages (**g**, **i**). n=3 per group, 3 technical replicates.

(**j**) Schematic of FITC Dextran lymphatic assay. FITC Dextran was injected into the rear footpad and popliteal lymph nodes were harvest 30 minutes after injection to measure FITC fluorescence intensity in popliteal lymph nodes. n=5 per group, 2 technical replicates.

P-values from two-sided Student's t-test (**b**, **c**, **e**, **g**, **i**, **j**). Error bars represent SEM (**b**, **c**, **e**, **g**, **i**, **j**).

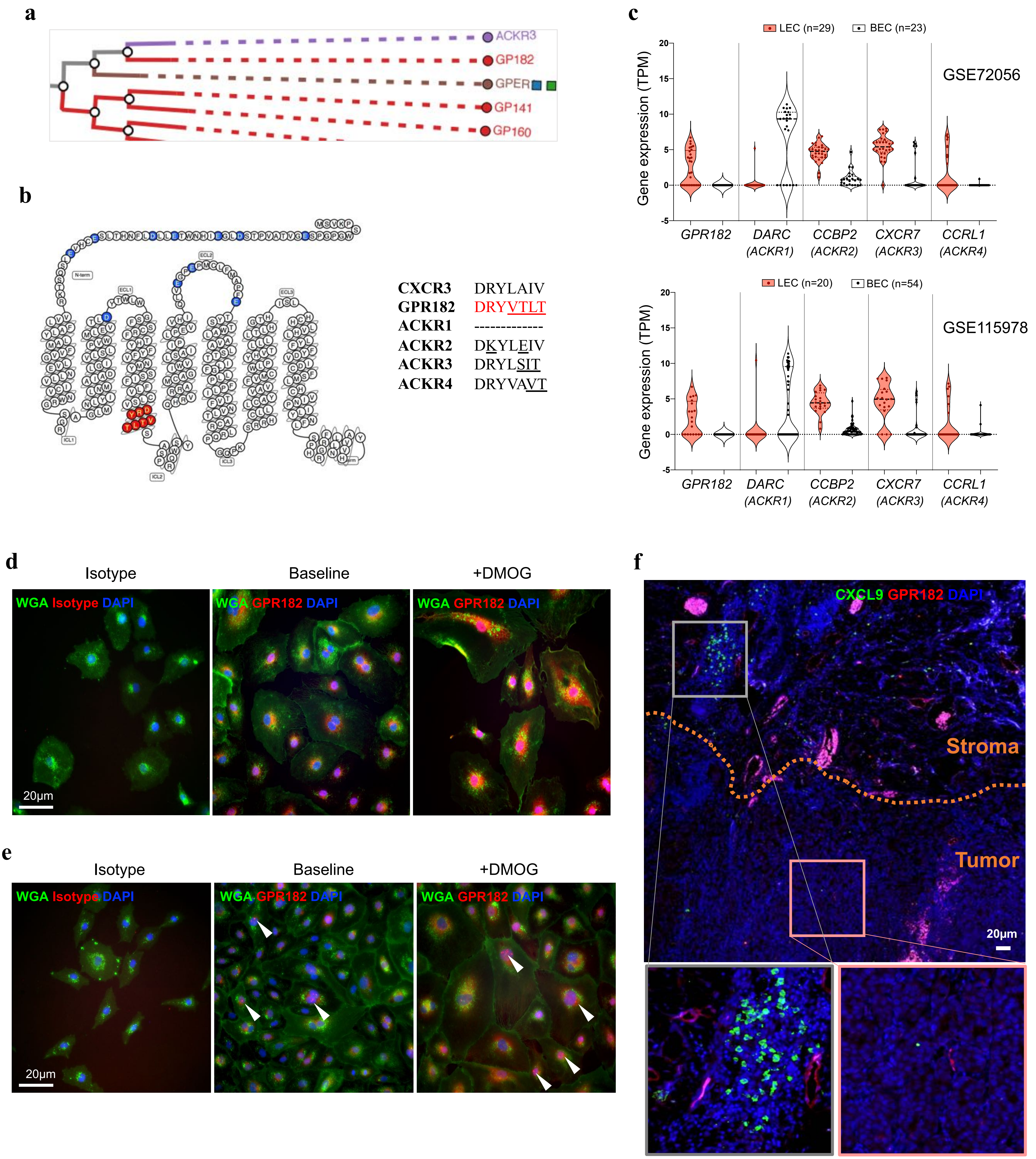
a
ACKR3
GP182
GPER
GP141
GP160
b
N-term
ECL1
ECL2
ECL3
ICL1
ICL2
ICL3
CXCR3 DRYLAIV
GPR182 DRYVTLT
ACKR1 -------------
ACKR2 DKYLEIV
ACKR3 DRYLSIT
ACKR4 DRYVAVT
c
LEC (n=29)
BEC (n=23)
GSE72056
Gene expression (TPM)
GPR182
DARC (ACKR1)
CCBP2 (ACKR2)
CXCR7 (ACKR3)
CCRL1 (ACKR4)
LEC (n=20)
BEC (n=54)
GSE115978
d
Isotype
Baseline
+DMOG
WGA Isotype DAPI
WGA GPR182 DAPI
20µm
e
Isotype
Baseline
+DMOG
WGA Isotype DAPI
WGA GPR182 DAPI
20µm
f
CXCL9 GPR182 DAPI
Stroma
Tumor
20µm

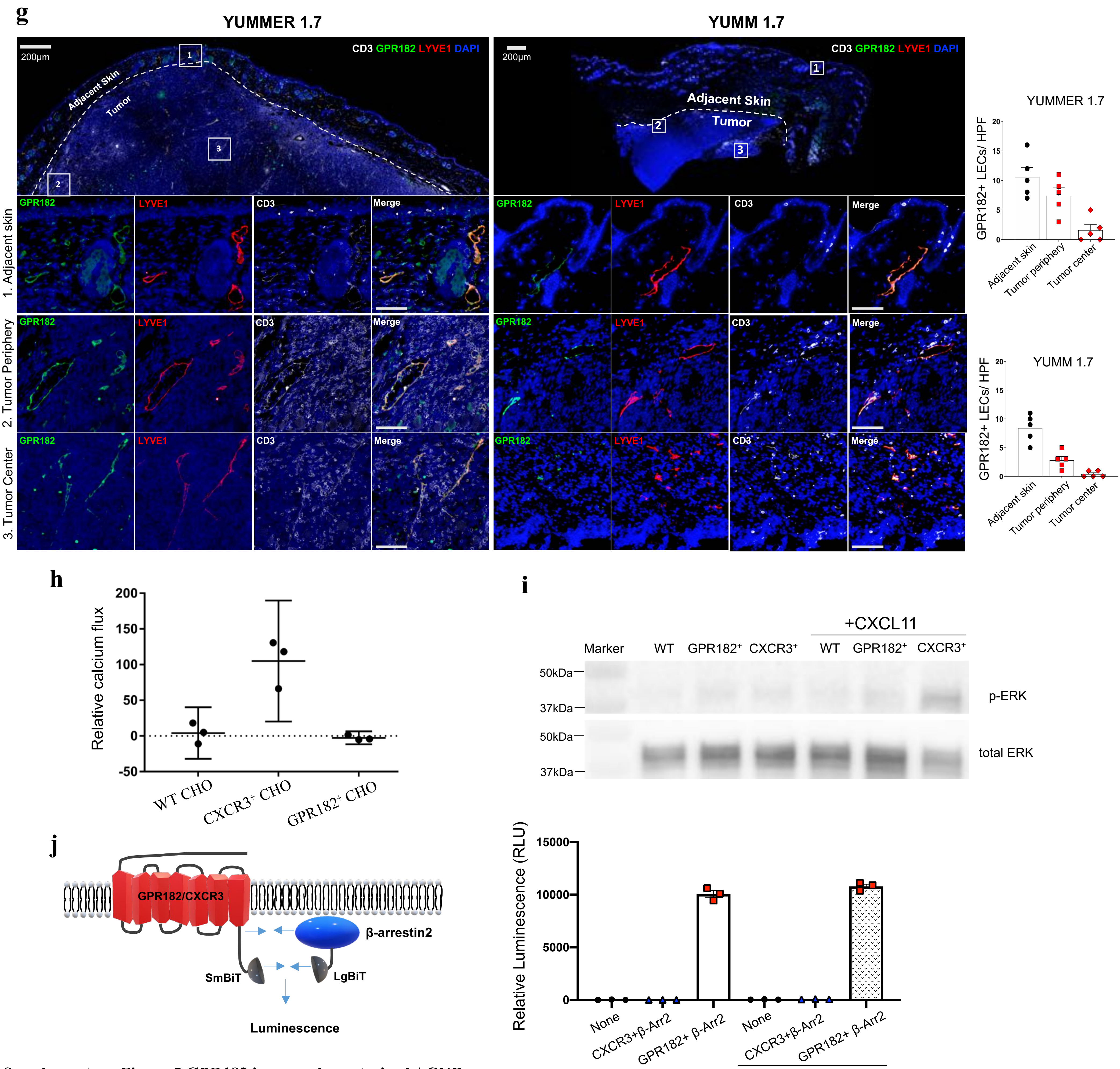

Supplementary Figure 5 GPR182 is an uncharacterized ACKR.

(**a**) Homologous tree of human GPR182 (GP182) to other GPCR molecules (www.gpcrdb.org).

(**b**) The amino acid (AA) sequence and secondary structure of human GPR182 protein (www.gpcrdb.org). The DRYLAIV motif in GPR182 is labeled in red and is aligned with other ACRK receptors. Chemokine receptor CXCR3 is used as a comparison.

(**c**) Analyses of two published datasets of human melanoma for the distributions of GPR182 and four known ACKRs in blood EC (BEC) and LEC.

(**d**) Human primary LECs were treated with DMOG overnight. Cells were fixed and stained for GPR182 expression.

(**e**) Human primary LECs were cultured with GPR182 mAb-AF647 at 37 degree for 1 hour. Cells were washed and followed with fixation and costaining with WGA. IF microscopy was used to locate GPR182 mAb.

(**f**) Human melanoma tissue was costained for CXCL9 and GPR182. The dash line separates tumor core (Tumor) from the peritumoral stroma (stroma).

(**g**) YUMMER1.7 and YUMM1.7 tumors from WT B6 mice were co-stained for mouse GPR182 , LYVE-1, and CD3. n = 5 per group.

(**h**, **i**) GPR182^{+} CHO and CXCR3^{+} CHO cells were stimulated by CXCL11 protein (100nM) for 3 minutes. Calcium flux (H) were measured. WT CHO served as a negative control. Each dot represents one independent assay. n = 3 independent assays. ERK phosphorylation was detected by western blot (I). Molecular weight markers were on the left side.

(**j**) NanoBiT analysis of the association between β-arrestin2 and GPR182/CXCR3 in response to CXCL11 binding. Data shown were luminescence values at 1 min after substrate addition. n = 3 per group.

Error bars represent SEM (**g**, **h**, **j**). Representative data from two (**d**-**j**) independent experiments.

Supplementary table 1 Expression and Function of Atypical Chemokine Receptor Family Members.

Receptor	Ligands	Expression	DRYLAIV Motif	Function	Knockout Mouse Phenotype
**ACKR1 (DARC)**	CCL2, 5, 7, 8, 11, 13, 14, 16, 17, 22; CXCL5, 6, 7, 8, 9, 11, 13	Erythrocytes, BECs	No Motif	Chemokine scavenging (erythrocytes), Chemokine transcytosis (BECs)	-Increased neutrophil recruitment in acute inflammation
**ACKR2 (D6)**	CCL2, 3, 3L1, 4, 5, 7, 8, 11, 13, 14, 17, 22	LECs, B cells, some macrophages	DKYLEIV	Chemokine scavenging	-Severe psoriatic pathology after TPA application
**ACKR3 (RDC1, CXCR7)**	CXCL11, 12	BECs and LECs, Stromal Cells, B cells	DRYLSIT	Chemokine scavenging, gradient shaping	-Prenatal lethality
**ACKR4 (CCRL1, CCX-CKR)**	CCL19, 21, 25; CXCL13	LECs, Keratinocytes, Thymic epithelium	DRYVAVT	Chemokine scavenging	-Impaired homeostatic homing of DCs from skin to draining lymph nodes
**GPR182**	chemokines broadly	LECs, ECs	DRYVTLT	Chemokine scavenging	-Increased myeloid cells; increase TIL infiltration

Abbreviations: lymphatic endothelial cells (LECs), blood endothelial cells (BECs), dendritic cells (DCs)
Table adapted from Bonechhi et al.[13], Griffith et al.[14], Le Mercier [19], Kwon et al [20], Mollica Poeta et al [34], and Bachelerie et al.[54].
