# Supplementary material for: GPR182 limits antitumor immunity via chemokine scavenging in mouse melanoma models
Source: Nat Commun. 2022 Jan 10;13:97. doi: 10.1038/s41467-021-27658-x (PMC8748779; doi:10.1038/s41467-021-27658-x)
Supplement: Supplementary file 3 — Reporting Summary [file 41467_2021_27658_MOESM3_ESM.pdf]

nature portfolio

Corresponding author(s): Yuwen Zhu

Last updated by author(s): 2021/11/08

Reporting Summary

Nature Portfolio wishes to improve the reproducibility of the work that we publish. This form provides structure for consistency and transparency in reporting. For further information on Nature Portfolio policies, see our Editorial Policies and the Editorial Policy Checklist.

Statistics

For all statistical analyses, confirm that the following items are present in the figure legend, table legend, main text, or Methods section.

n/a	Confirmed	
☐	☒	The exact sample size (*n*) for each experimental group/condition, given as a discrete number and unit of measurement
☐	☒	A statement on whether measurements were taken from distinct samples or whether the same sample was measured repeatedly
☐	☒	The statistical test(s) used AND whether they are one- or two-sided<br>*Only common tests should be described solely by name; describe more complex techniques in the Methods section.*
☐	☒	A description of all covariates tested
☒	☐	A description of any assumptions or corrections, such as tests of normality and adjustment for multiple comparisons
☐	☒	A full description of the statistical parameters including central tendency (e.g. means) or other basic estimates (e.g. regression coefficient) AND variation (e.g. standard deviation) or associated estimates of uncertainty (e.g. confidence intervals)
☐	☒	For null hypothesis testing, the test statistic (e.g. *F*, *t*, *r*) with confidence intervals, effect sizes, degrees of freedom and *P* value noted<br>*Give P values as exact values whenever suitable.*
☒	☐	For Bayesian analysis, information on the choice of priors and Markov chain Monte Carlo settings
☒	☐	For hierarchical and complex designs, identification of the appropriate level for tests and full reporting of outcomes
☐	☒	Estimates of effect sizes (e.g. Cohen's *d*, Pearson's *r*), indicating how they were calculated

Our web collection on statistics for biologists contains articles on many of the points above.

Software and code

Policy information about availability of computer code

Data collection	Transcriptome data from TCGA Research Network was collected from: www.cbioportal.org. Human melanoma single cell data were collected from published datasets GSE115978 and GSE72056.
Data analysis	The software used to analyze the data include: GraphPad Prism 7.0 and 8.0 (GraphPad Software); STATA 15 (StataCorp); FlowJo 10 (FlowJo, LLC), SlideBook (Verson 6, Intelligent Imaging Inc), and GeneSys software (Syngene).

For manuscripts utilizing custom algorithms or software that are central to the research but not yet described in published literature, software must be made available to editors and reviewers. We strongly encourage code deposition in a community repository (e.g. GitHub). See the Nature Portfolio guidelines for submitting code & software for further information.

Data

Policy information about availability of data

All manuscripts must include a data availability statement. This statement should provide the following information, where applicable:

- Accession codes, unique identifiers, or web links for publicly available datasets
- A description of any restrictions on data availability
- For clinical datasets or third party data, please ensure that the statement adheres to our policy

We used public databases to evaluate GPR182 transcript in human melanoma. The scRNAseq datasets presented in Fig. 1a and 1b were derived from GSE115978 and GSE72056. Data for GPR182 expression and lymphatic score in metastatic melanoma (Supplementary Fig. 1d) was extracted from the TCGA database (https://portal.gdc.cancer.gov). Other relevant data supporting the key findings of this study are available within the article and its Supplementary Information files. All numerical source data underlying Figs. 1-8 and Supplementary Figs. 1-5 are provided as a Source Data file.

Field-specific reporting

Please select the one below that is the best fit for your research. If you are not sure, read the appropriate sections before making your selection.

☒ Life sciences ☐ Behavioural & social sciences ☐ Ecological, evolutionary & environmental sciences

For a reference copy of the document with all sections, see nature.com/documents/nr-reporting-summary-flat.pdf

Life sciences study design

All studies must disclose on these points even when the disclosure is negative.

Sample size	No statistical method was used to determine the sample size for human tumor immunoflourescence analysis as the samples used represented those available with paired adjacent normal tissue. We used a total of 15 human melanoma samples with paired adjacent normal tissue. To generalize our findings with a larger sample size, gene expression data was also used from publicly available datasets. For mice studies, we targeted a sample size of 5-10 mice per group and specify the exact sample size for each experiment. This is a standard sample size for these experiments within the ethical considerations of animal use protocol at our institution.
Data exclusions	No data were excluded from the analyses.
Replication	All experiments were repeated and the number of repeats and technical replicates per experiment are including in the text and/or figure legends.
Randomization	For knockout mice studies, mice were grouped by genotype and treated equally. For in vivo treatments, mice were randomly allocated between treatment and control groups prior to the first treatment. For in vitro experiments, we randomized groups for control or intervention treatment.
Blinding	For most tumor experiments, we have managed to have the leading scientist to perform tumor inoculation and therapeutic treatment while having the tumor measurement by our technician or a different assisting postdoc. Sometime, blinding was not possible due to treatment schedules and personnel limitations. For other in vitro experiments, blinding is impossible and there is no step that can introduce personal bias.

Reporting for specific materials, systems and methods

We require information from authors about some types of materials, experimental systems and methods used in many studies. Here, indicate whether each material, system or method listed is relevant to your study. If you are not sure if a list item applies to your research, read the appropriate section before selecting a response.

Materials & experimental systems

n/a	Involved in the study	
☐	☒	Antibodies
☐	☒	Eukaryotic cell lines
☒	☐	Palaeontology and archaeology
☐	☒	Animals and other organisms
☐	☒	Human research participants
☒	☐	Clinical data
☒	☐	Dual use research of concern

Methods

n/a	Involved in the study	
☒	☐	ChIP-seq
☐	☒	Flow cytometry
☒	☐	MRI-based neuroimaging

Antibodies

Antibodies used

For flow cytometry staining
CD16/32 (anti-FcyIII/II receptor); Clone 93; BioLegend; Cat# 101302
CD45.2; Clone 104; BioLegend; BV605 Cat# 109841
CD19; Clone 6D5; BioLegend; APC; Cat# 115502
CD3; Clone 17A2; BioLegend; APC; Cat# 100236
NK1.1; Clone PK136: BioLegend; BV510; Cat# 108737
CD11b; Clone M1/70; Biolegend; PE; Cat# 101208
CD11c; Clone N418; BioLegend; BV421; Cat# 117343
I-Ab; Clone AF6-120.1; BioLegend; PE/Cy7; Cat# 116420
Ly-6G; Clone IA8; BioLegend; FITC; Cat# 127606
Ly-6C; Clone HK1.4; BioLegend; perCP/Cy5.5; Cat# 128016
F4/80; Clone BM8; BioLegend; BV711; Cat# 123147
CD8a; Clone 53-6.7; BioLegend; perCP/Cy5.5; Cat# 100734
CD4; Clone RM4-5; BioLegend; BV510; Cat# 100510
CD44; Clone IM-7; BioLegend; PE/Cy7; Cat# 103030

CD62L; Clone MEL-14; BioLegend; BV711; Cat# 104445
PD1; Clone RMPI-30; BioLegend; BV421; Cat# 109121
FoxP3; Clone MF-14; BioLegend; PE; Cat# 126404
H-2Kb bound SIINFEKL; Clone 25-D1.16; BioLegend; PE; Cat# 141604
GzmB; Clone GB11; BioLegend; FITC; Cat# 515403
IFN-γ; Clone XMG1.2; BioLegend; PE; Cat# 505808
4-1BB; Clone 17B5; BioLegend; PE; Cat# 106106
CXCR3; Clone CXCR3-173; BioLegend; PE/Cy7; Cat# 126516
Ghost Dye Red 780; Tonbo Biosciences; Cat# 13-0865-T100

In vivo treatments:
anti-CD4; Clone GK1.5; BioXcell; Cat# BE0003-1
anti-CD8b; Clone 53-5.8; BioXcell; Cat# BE0223
anti-CXCR3; Clone CXCR3-173; BioXcell; Cat# BE0249
anti-PD1; Clone RMP1-14; BioXcell; Cat# BE0146
anti-CTLA-4; Clone 9D9; BioXcell; Cat# BE0164

IF staining:
anti-human GPR182 (1:800); Clone PA5-110928; Invitrogen; Cat# PA5-110928
anti-human CD31; Clone JC/70A; Thermo Fischer; Cat# MA5-13188
anti-human PDPN: Clone D2-40; Agilent; Cat# M361929-2
anti-mouse IgG (H+L) secondary antibody (Cy3); Invitrogen; Cat# A10521
anti-rabbit IgG (H+L) secondary antibody (Cy5); Invitrogen; Cat# A10523
anti-mouse CD31; Clone 390; BioLegend; Cat# 102402
anti-mouse CD3e; Clone 145-2C11; BioLegend; APC; Cat# 100312
anti-DYKDDDDK Tag; Clone L5; BioLegend; Cat# 637301
anti-GPR182 (ADMR); AF 647-conjugated; Clone 528563; R&D Systems; Cat# FAB10293R-100UG
anti-caveolin-1; Clone D46G3; Cell Signaling; Cat# 3267
anti-clathrin-HC; Clone D3C6; Cell Signaling; Cat# 4796
anti-LAMP1; Clone D2D11; Cell Signaling; Cat# 9091S
anti-human Prox-1; Clone D2J6J; Cell Signaling; Cat# 14963S

Validation

Most of these antibodies were purchased from Biolegend, Cell Signaling, and BioXcell. They have been used in many literature and validated from a lot publications. Companies often validate each batch before selling. Our lab routinely validates new antibodies via flow cytometry and western blot, such as antibodies for human GPR182.
Flow cytometry:
Antibodies from Biolegend:
CD16/32 (anti-FcγIII/II receptor); Clone 93; (https://www.biolegend.com/en-us/search-results/purified-anti-mouse-cd16-32-antibody-190);
CD45.2; Clone 104; (https://www.biolegend.com/en-us/products/brilliant-violet-605-anti-mouse-cd45-2-antibody-9695);
CD19; Clone 6D5; (https://www.biolegend.com/en-us/products/purified-anti-mouse-cd19-antibody-1532);
CD3; Clone 17A2; (https://www.biolegend.com/en-us/products/apc-anti-mouse-cd3-antibody-8055);
NK1.1; Clone PK136: (https://www.biolegend.com/en-us/products/brilliant-violet-510-anti-mouse-nk-1-1-antibody-8615);
CD11b; Clone M1/70; (https://www.biolegend.com/en-us/products/pe-anti-mouse-human-cd11b-antibody-349);
CD11c; Clone N418; (https://www.biolegend.com/en-us/products/brilliant-violet-421-anti-mouse-cd11c-antibody-7149);
I-Ab; Clone AF6-120.1; (https://www.biolegend.com/en-us/products/pe-cyanine7-anti-mouse-i-ab-antibody-6623);
Ly-6G; Clone IA8; (https://www.biolegend.com/en-us/search-results/fitc-anti-mouse-ly-6g-antibody-4775);
Ly-6C; Clone HK1.4; (https://www.biolegend.com/en-us/products/apc-anti-mouse-ly-6c-antibody-6047);
F4/80; Clone BM8; (https://www.biolegend.com/en-us/products/brilliant-violet-711-anti-mouse-f4-80-antibody-10705);
CD8a; Clone 53-6.7; (https://www.biolegend.com/en-us/products/percp-cyanine5-5-anti-mouse-cd8a-antibody-4255);
CD4; Clone RM4-5; (https://www.biolegend.com/en-us/products/fitc-anti-mouse-cd4-antibody-480);
CD44; Clone IM-7; (https://www.biolegend.com/en-us/products/pe-cyanine7-anti-mouse-human-cd44-antibody-3932);
CD62L; Clone MEL-14; (https://www.biolegend.com/en-us/products/brilliant-violet-711-anti-mouse-cd62l-antibody-10317);
PD1; Clone RMPI-30; (https://www.biolegend.com/en-us/products/brilliant-violet-421-anti-mouse-cd279-pd-1-antibody-14324);
FoxP3; Clone MF-14; (https://www.biolegend.com/en-us/search-results/pe-anti-mouse-foxp3-antibody-4660);
H-2Kb bound SIINFEKL; Clone 25-D1.16; (https://www.biolegend.com/en-us/products/pe-anti-mouse-h-2kb-bound-to-siinfekl-antibody-7247);
GzmB; Clone GB11; (https://www.biolegend.com/en-us/products/fitc-anti-human-mouse-granzyme-b-antibody-6066);
IFN-γ; Clone XMG1.2; (https://www.biolegend.com/en-us/products/pe-anti-mouse-ifn-gamma-antibody-997);
4-1BB; Clone 17B5; (https://www.biolegend.com/en-us/products/pe-anti-mouse-cd137-antibody-51);
CXCR3; Clone CXCR3-173; (https://www.biolegend.com/en-us/products/pe-cyanine7-anti-mouse-cd183-cxcr3-antibody-6169);

Abs from Tonbo Biosciences;
Ghost Dye Red 780; Cat# 13-0865-T100 (http://tds.tonbobio.com/tds-13-0865.pdf);

IF staining:
anti-human GPR182 (1:800); Clone PA5-110928; (https://www.thermofisher.com/order/genome-database/dataSheetPdf?producttype=antibody&productsubtype=antibody_primary&productId=PA5-110928&version=180);
anti-human CD31; Clone JC/70A; (https://www.thermofisher.com/order/genome-database/dataSheetPdf?producttype=antibody&productsubtype=antibody_primary&productId=MA5-13188&version=180);
anti-human PDPN: Clone D2-40; (https://www.agilent.com/cs/library/packageinsert/public/305832EN_04.pdf);
anti-mouse CD31; Clone 390; (https://www.biolegend.com/en-us/products/purified-anti-mouse-cd31-antibody-123);

anti-mouse CD3e; Clone 145-2C11; (https://www.biolegend.com/en-us/products/apc-anti-mouse-cd3epsilon-antibody-21);
anti-GPR182 (ADMR); AF 647-conjugated; Clone 528563; (https://resources.rndsystems.com/pdfs/datasheets/mab10293.pdf?v=20211107&_ga=2.236188676.449541751.1636303166-467470968.1636303165);
anti-caveolin-1; Clone D46G3; (https://www.cellsignal.com/products/primary-antibodies/caveolin-1-d46g3-xp-rabbit-mab/3267);
anti-clathrin-HC; Clone D3C6; (https://www.cellsignal.com/products/primary-antibodies/clathrin-heavy-chain-d3c6-xp-rabbit-mab/4796);
anti-LAMP1; Clone D2D11; (https://www.cellsignal.com/products/primary-antibodies/lamp1-d2d11-xp-rabbit-mab/9091);
anti-human Prox-1; Clone D2J6J; (https://www.cellsignal.com/products/primary-antibodies/prox1-d2j6j-rabbit-mab/14963);

Antibodies from BioXcell for in vivo usage:
Anti-mouse CD8β mAb (https://d2a7cdyquyl45u.cloudfront.net/tds-sheets/BE0223-tds.pdf), anti-mouse CD4 mAb (https://d2a7cdyquyl45u.cloudfront.net/tds-sheets/BE0003-1-tds.pdf), anti-mouse CTLA-4 mAb (https://d2a7cdyquyl45u.cloudfront.net/tds-sheets/BE0164-tds.pdf), anti-mouse TIGIT mAb (https://d2a7cdyquyl45u.cloudfront.net/tds-sheets/BE0274-tds.pdf), anti-mouse PD-1 mAb (https://d2a7cdyquyl45u.cloudfront.net/tds-sheets/BE0146-tds.pdf)

Eukaryotic cell lines

Policy information about cell lines

Cell line source(s)

B16 melanoma cells were originally obtained from ATCC (ATCC.org).
B16-OVA cells, , available at Sigma-Aldrich, were acquired from Dr. Lieping Chen (Yale University, New Haven, CT).
YUMM1.7 and YUMMER1.7 cells, acquired from Dr. Mayumi Fujita (University of Colorado, Auora, CO) , are developed by Dr. Marcus Bosenberg at Yale. YUMM1.7 cell can be obtained from ATCC (ATCC.org)..
HEK293 and CHO cells were originally obtained from ATCC (ATCC.org).

Authentication

All cells are grown and frozen a large batch from ATCC or from collaborators. Tumor cells were authenticated by morphology, proliferation in vitro and tumorigenticity in vivo. The expression of OVA antigen in B16-OVA was confirmed by flow cytometry staining with a Kb-OVA antibody. We limit cell passages and only use cell lines within 6 passages from ATCC if possible.

Mycoplasma contamination

All cell lines used are negative for mycoplasma contamination.

Commonly misidentified lines
(See ICLAC register)

None of the cell lines used in this study are listed in the ICLAC database.

Animals and other organisms

Policy information about studies involving animals; ARRIVE guidelines recommended for reporting animal research

Laboratory animals

GPR182-/- mice were bred in-house using Gpr182tm2a(KOMP)Wtsi/+ (knockout first/promoter driven) mice which were generated and obtained from the Knockout Mouse Project (KOMP) Repository (www.komp.org). Both WT littermates and purchased C57BL/6 mice were used as wild type controls. OT-1 T cell receptor (TCR) transgenic mice were obtained from the Jackson Laboratory and crossed with CD45.1+/+ mice to obtain CD45.1+ OT-1 mice. Experiments used age and sex matched WT C57BL/6j and GPR182-/- mice between 8 and 12 weeks of age. We used both male and female mice for the B16 and B16-OVA tumor models. Only male mice were used for the YUMM1.7 and YUMMER1.7 tumor models as they were derived from male mice.

Wild animals

This study did not involve wild animals.

Field-collected samples

This study did not involve samples collected from the field.

Ethics oversight

All animal care procedures and experiments were approved by the Institutional Animal are and Use Committee at the University of Colorado Anschutz Medical Campus.

Note that full information on the approval of the study protocol must also be provided in the manuscript.

Human research participants

Policy information about studies involving human research participants

Population characteristics

Population characteristics are not included in this study.

Recruitment

No participants were recruited for this study.

Ethics oversight

This study was exempt from human subject research ethics oversight as no clinical or identifiable data was used.

Note that full information on the approval of the study protocol must also be provided in the manuscript.

Flow Cytometry

Plots

Confirm that:

☒ The axis labels state the marker and fluorochrome used (e.g. CD4-FITC).

☒ The axis scales are clearly visible. Include numbers along axes only for bottom left plot of group (a 'group' is an analysis of identical markers).

☒ All plots are contour plots with outliers or pseudocolor plots.

☒ A numerical value for number of cells or percentage (with statistics) is provided.

Methodology

Sample preparation	Tumors tissues were resected and weighed before processing. Following mechanical homogenization, the tissue was resuspended in RPMI1640 media containing Liberase DM (Roche Diagnostics Corporation). Tumors were digested for 30 minutes at 37°C and subsequently passed through a cell strainer. For isolation of single-cell suspensions from lymph nodes, tissue was first disrupted with 18-guauge needles prior to digestion.
Instrument	Flow cytometric analysis was conducted on BD FACS Calibur and Beckman Coulter CytoFlex S.
Software	Data were analyzed by FlowJo software (FlowJo, LLC).
Cell population abundance	Not applicable. Cell sorting was not performed.
Gating strategy	The gating strategy used for immune cell populations can be found in Supplemental Figure 2. Single cells were distinguished from debris via ssc and fsc gating and live cells were identified by ghost dye. CD45.2 staining was used to identify Immune cells. T cells (CD3, CD4, and CD8) were gated out for further phenotypical analysis. Non-T cells were analyzed with surface markers to identify NK cells, DCs, macrophages and MDSCs.

☒ Tick this box to confirm that a figure exemplifying the gating strategy is provided in the Supplementary Information.
